# Effect of basic psychological satisfaction needs on resilience in patients with first acute myocardial infarction: the mediating role of family resilience and hope

**DOI:** 10.3389/fpsyt.2025.1670046

**Published:** 2025-09-29

**Authors:** Hong Ding, Liyun Miao, Yali Bai, Yan Wang

**Affiliations:** Department of Cardiology Ward II, Xinxiang Central Hospital, The Fourth Clinical College of Henan Medical University, Xinxiang, Henan, China

**Keywords:** acute myocardial infarction, basic psychological need satisfaction, family resilience, hope, mediation analysis

## Abstract

**Background:**

Psychological resilience is key to coping with adversity, stress buffering, and trauma. Acute myocardial infarction (AMI), a major life event, triggers severe psychological stress, especially in first-time patients facing heightened adversity. This diminishes resilience and worsens the prognosis. Evidence links basic psychological need satisfaction to resilience, but the mediating roles of family resilience and hope remain untested. This study aimed to explore the influence of psychological satisfaction needs on the psychological resilience of patients with first-time AMI, and to investigate the chain intermediary role of family resilience and hope in it.

**Methods:**

This cross-sectional study was conducted from June 2023 to June 2025. Patients with first-time AMI and treated at our hospital were enrolled via convenience sampling. Data were collected using Basic Psychological Needs Scales (BPNS), Basic Psychological Needs Scales (BPNS), Family Resilience Assessment Scale (FRAS) and the Herth Hope Index (HHI). Statistical analysis included an independent sample T test, Pearson correlation analysis, linear regression and self-help intermediary analysis.

**Results:**

A total of 179 first-time AMI patients showed mean scores of (62.95 ± 5.71) on the CD-RISC, (110.35 ± 14.00) on the BPNS, (37.28 ± 9.87) on the FRAS, and (24.63 ± 5.93) on the HHI. Significant positive correlations were observed between CD-RISC scores and BPNS, FRAS, and HHI scores (P < 0.05). The total effect of basic psychological needs satisfaction on psychological resilience was significant (β = 0.273, P < 0.001), with a significant direct predictive effect. Basic psychological needs satisfaction positively predicted family resilience (β = 0.489, P < 0.001) and hope (β = 0.262, P < 0.001). Both family resilience (β = 0.211, P < 0.001) and hope (β = 0.273, P < 0.001) demonstrated significant positive effects on psychological resilience. Path analysis confirmed four significant mediation pathways (all 95% CIs excluded zero).

**Conclusion:**

Family resilience and hope mediate basic psychological needs and psychological resilience in first-time AMI patients. Clinical care should integrate family-community resources to enhance social support and companionship, aiming to boost patients' resilience and promote prognosis.

## 1 Introduction

Acute myocardial infarction (AMI), defined as ischemic myocardial necrosis resulting from acute coronary occlusion and insufficient blood perfusion, typically manifests as crushing chest pain with high mortality and disability rates (1). The development of AMI is closely associated with emotional stress and behavioral patterns, establishing a vicious cycle through interactions between psychological-behavioral and somatic factors (2).

Psychological resilience refers to an individual's capacity to process severe stressors and adverse events (3). This construct constitutes a critical element in mental health cultivation and psychological crisis intervention. Studies confirm that resilience functions as a protective factor against psychological distress, fostering stress resistance and facilitating positive adaptation to adversity (4, 5). Given the sudden onset, life-threatening nature, and lack of coping experience, first-time AMI patients frequently develop severe psychological shock due to intense fear of poor prognosis, significantly reducing resilience levels (6). Diminished resilience not only amplifies psychological distress but also directly compromises treatment adherence and rehabilitation progress, potentially elevating risks of major adverse cardiovascular events (MACE) (7). Consequently, assessment of psychological resilience and its influencing factors in first-time AMI patients carries vital clinical significance (8). This approach enables disruption of the psychosomatic vicious cycle, development of precise psychological interventions, and ultimately improvement in patients' quality of life and disease prognosis.

The basic psychological needs theory posits that humans possess three fundamental psychological needs: autonomy, relatedness, and competence (9). When autonomy needs are fulfilled, patients more effectively engage in shared treatment decision-making, enhancing their sense of control during clinical processes. This is essential for developing psychological resilience and facilitating positive adaptation to health challenges. The satisfaction of belongingness needs fosters robust social support, which helps to buffer the stress of illness, sustain psychological resilience, and mobilize external resources (10). While the satisfaction of competence needs can improve self-efficacy, helping patients rebuild their confidence and promoting their internal resources to cope with adversity. Consequently, satisfaction of these three basic psychological needs correlates significantly with mental health and establishes the foundation for building and maintaining psychological resilience (11). Therefore, the state of basic psychological need satisfaction in first-time AMI patients not only associates with their resilience levels but also critically influences psychosomatic recovery trajectories. Nevertheless, the precise pathways and mechanisms mediating these effects warrant further exploration.

Family resilience refers to a family system's capacity to achieve healthy adaptation when confronting adversity or stress (12). Strong family resilience mitigates patients' negative psychological states such as anxiety and depression, exerting profound positive impacts on their physical and mental health. Hope, defined as a positive psychological state enabling individuals to believe in overcoming life challenges and achieving personal goals, empowers patients to establish clearer objectives and pursuits (13). Elevated hope levels enhance psychological resilience, while improved resilience reciprocally sustains hope.

Therefore, both family resilience and hope significantly influence patients' basic psychological need satisfaction. Building on this theoretical framework, this study aims to investigate the impact of basic psychological needs satisfaction on resilience among first-time AMI patients, examining the chain mediation effects of family resilience and hope. These findings will provide theoretical foundations and identify potential intervention targets for enhancing resilience in this population.

## 2 Methods

### 2.1 Participants

From June 2023 to June 2025, patients with AMI treated in our hospital were selected by convenient sampling as the research object.

Inclusion criteria: (1) a confirmed diagnosis of AMI, including both ST-segment elevation myocardial infarction (STEMI) and non-ST-segment elevation myocardial infarction (NSTEMI), based on standard clinical diagnostic criteria (14); (2) first onset; (3) have a certain understanding ability, can communicate in language or in writing; (4) all patients underwent coronary angiography and percutaneous coronary intervention.

Exclusion criteria: (1) persons with mental illness, audio-visual impairment or severe cognitive impairment; (2) malignant tumor or severe heart failure and respiratory failure; (3) previous history of mental illness such as depression and anxiety; (4) the clinical data are incomplete or they are still unwilling to cooperate with this researcher after explanation.

### 2.2 Research methods

#### 2.2.1 Clinical data collection

Clinical data were collected from patient interviews and medical records. Collected variables included age, gender, education level, marital status, family per capita monthly income, source of expenses, whether they are complicated with hypertension and BMI. The number of people to be investigated should be 10~15 times that of the items included in the survey. There are 15 items in this study, and 150~225 cases should be investigated. Considering the loss rate of 10%~30%, the sample size is at least 165 cases, and the sample size of this study is 186 cases.

#### 2.2.2 investigation of psychological resilience

The Connor-Davidson Resilience Scale (CD-RIS) was used to evaluate the mental resilience of all patients. This scale contains three factors, namely, resilience, optimism and strength, and 10 items, each of which is 0–4 points, with 0 indicating that this is not the case at all and 4 indicating that it is almost always the case. The higher the score, the higher the psychological resilience (15).

#### 2.2.3 investigation of basic psychological needs

The basic psychological needs scales (BPNS) were used to evaluate the basic psychological needs of all patients, including autonomous needs, competence needs and belonging needs, with 21 items. Likert 7-level scoring method is adopted, and "complete non-conformity" and "complete conformity" are scored 1–7 respectively, with a total score of 21-147. The higher the score, the higher the degree of demand satisfaction (16).

#### 2.2.4 investigation of family resilience

The family resilience assessment scale (FRAS) was used to evaluate the family resilience of all patients. The scale included 10 dimensions, including dilemma interpretation, forward-looking, excellent life, problem solving, intimacy and harmony, social support, orderly, emotional sharing, clear communication and cooperation and coordination, with 49 items. Likert's 5-level score was used, ranging from "non-conformity" to "conformity" (17).

#### 2.2.5 investigation of hope

The Herth Hope Index (HHI) was used to evaluate the hope level of all patients, which consisted of 12 items in 3 dimensions. The dimensions of "temporary attitude and future (T)" respectively include items 1, 2, 6 and 11. The dimension of "positive readiness and expectancy (P)" includes items 4, 7, 10 and 12; And the "inter-connectedness (I)" dimension includes items 3, 5, 8 and 9. The scale adopts Likert1~4 scale, and the score range is 0~48. The higher the score, the higher the hope level (18).

### 2.3 Quality control

All questionnaires were distributed and collected by researchers. Before filling in, the purpose of this study was explained to patients, and all respondents agreed to fill in. In the process of filling in, unified instructions were used, and all questionnaires were collected on the spot.

### 2.4 Data processing

All statistical analyses were conducted using SPSS version 24.0 (IBM Corp., Armonk, NY, USA). Descriptive statistics were used to summarize the demographic and clinical characteristics. Categorical variables are reported as frequencies and percentages, while continuous variables were tested for normality using the Shapiro–Wilk test. Normally distributed data are expressed as mean ± standard deviation (± SD). Correlation analyses were conducted to assess the associations between key variables. To evaluate the mediation effects, a bootstrap resampling method with 1,000 iterations and bias-corrected confidence intervals was employed. A mediation effect was considered statistically significant if the 95% confidence interval did not include zero.

## 3 Results

### 3.1 General information of patients

In this study, a total of 186 questionnaires were distributed, 7 invalid questionnaires were excluded, and 179 questionnaires were finally recovered, with an effective rate of 96.24%. The general information of all subjects is shown in **Table 1**.

**Table 1:** General information in 179 patients with first-time AMI.

Variables		Cases/ $\bar{x}$ ± SD
Age (year)	≥60	84 (46.93)
	<60	95 (53.07)
Gender (n,%)	Male	92 (51.40)
	Female	87 (48.60)
Degree of education (n,%)	Junior high school and below	49 (27.37)
	High school and above	130 (72.63)
Marital status (n,%)	Unmarried	40 (22.35)
	Married	84 (46.93)
	Divorce	42 (23.46)
	Spouse	13 (7.26)
Religious beliefs (n,%)	Yes	37 (20.67)
	No	142 (79.33)
Average family monthly income (n,%)	<2000 yuan	31 (17.32)
	2000~4000 yuan	85 (47.49)
	>4000 yuan	63 (35.20)
Source of cost (n,%)	New farming	72 (40.22)
	Employee medical insurance	69 (38.55)
	Self-pay	38 (21.23)
Whether complicated with hypertension (n,%)	Yes	61 (34.08)
	No	118 (65.92)
BMI(kg/m^2^)		24.38 ± 3.86
Killip grade (n,%)	I	37 (20.67)
	II	68 (37.99)
	III	74 (41.34)
Length of hospital stay (d)		16.59 ± 4.16

BMI, body mass index.

### 3.2 The scores of CD-RIS, BPNS, FRAS and HHI in 179 patients with first-time AMI

A total of 179 first-time AMI patients showed mean scores of (62.95 ± 5.71) on the CD-RISC, (110.35 ± 14.00) on the BPNS, (37.28 ± 9.87) on the FRAS, and (24.63 ± 5.93) on the HHI. The scores of each dimension of each scale are shown in **Table 2**.

**Table 2:** Scores of CD-RIS, BPNS, FRAS and HHI in 179 patients with first-time AMI.

Item		Scare ( $\bar{x}$ ± S, points)
CD-RIS	Tenacity	27.12 ± 1.81
	Optimism	16.89 ± 1.92
	Strength	18.94 ± 1.98
BPNS	Independent needs	40.31 ± 5.38
	Competency needs	38.76 ± 4.52
	Attribution needs	31.28 ± 4.10
FRAS	Dilemma solution	3.98 ± 0.84
	Forward-looking	3.54 ± 0.84
	Excellent life	3.61 ± 0.71
	Problem solving	3.75 ± 0.78
	Intimacy and harmony	3.84 ± 0.83
	Social support	3.45 ± 1.13
	Order well	3.72 ± 1.09
	Emotional sharing	3.85 ± 1.02
	Clear communication	3.83 ± 0.93
	Cooperation and coordination	3.71 ± 1.01
HHI	T	7.63 ± 1.88
	P	8.69 ± 1.94
	I	8.31 ± 2.11

T, temporary attitude and future; P, positive readiness and expectancy; I, interconnectedness.

### 3.3 Correlation analysis of CD-RIS, BPNS, FRAS and HHI in patients with first-time AMI

There is a positive correlation between CD-RIS and BPNS, FRAS and HHI in patients (P < 0.001), as shown in **Table 3**.

**Table 3:** Correlation analysis of CD-RIS, BPNS, FRAS and HHI in patients with first-time AMI.

Item	CD-RIS	BPNS	FRAS	HHI
CD-RIS	1			
BPNS	0.513^**^	1		
FRAS	0.514^**^	0.489^**^	1	
HHI	0.541^**^	0.504^**^	0.622^**^	1

CD-RIS, the Connor-Davidson Resilience Scale; BPNS, basic psychological needs scales; FRAS, Family Resilience Assessment Scale; T, temporary attitude and future; P, positive readiness and expectancy; I, interconnectedness.

^**^P<0.001.

### 3.4 Analysis of intermediary effect

The total effect of basic psychological satisfaction needs of patients with first-time AMI is significant (β=0.273, P < 0.001), and the direct prediction of psychological resilience is significant. Basic psychological satisfaction can positively predict family resilience (β=0.489, P < 0.001) and hope level (β=0.262, P < 0.001). At the same time, both family resilience and hope level can positively predict patients' psychological resilience (β = 0.211, 0.273; P < 0.001), as shown in **Table 4**.

**Table 4:** Regression analysis of the mediating effect of psychological resilience, basic psychological satisfaction needs, family resilience and hope.

Item	FRAS			HHI			CD-RIS		
	SE	*t*	*β*	SE	*t*	*β*	*SE*	*t*	*β*
Constant	6.328	-0.087	–	3.276	0.415	–	3.293	-0.06	–
BPNS	0.057	6.041**	0.489	0.034	3.275**	0.262	0.035	3.126**	0.273
FRAS				0.048	6.169**	0.494	0.056	2.188**	0.211
HHI							0.094	2.803**	0.273
*R²*	0.243			0.438			0.397		
*F*	0.336			0.428			0.382		

CD-RIS, the Connor-Davidson Resilience Scale; BPNS, basic psychological needs scales; FRAS, Family Resilience Assessment Scale; T, temporary attitude and future; P, positive readiness and expectancy; I, interconnectedness.

^**^P<0.001.

### 3.5 Mediation effect test

**Figure 1:**
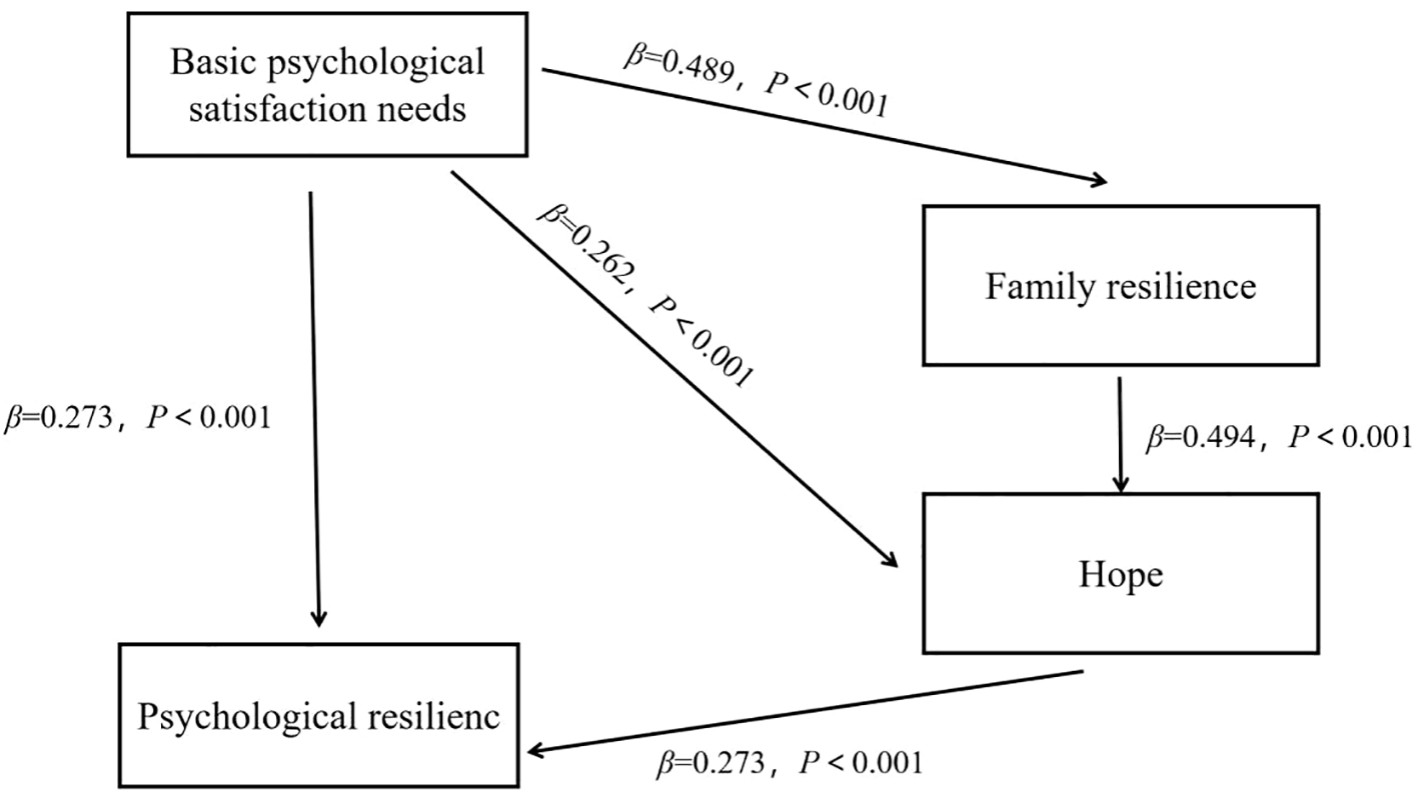
Chain mediation model of basic psychological satisfaction needs, psychological resilience, family resilience and hope level of patients with first-time AMI.

An intermediary pathway analysis is established to explore the indirect influence of basic psychological satisfaction needs on psychological resilience. The tested pathways included (1) Basic psychological satisfaction needs→Psychological resilience, (2) Basic psychological satisfaction needs→Family resilience→Psychological resilience, (3) Basic psychological satisfaction needs→Hope→Psychological resilience, and (4) Basic psychological satisfaction needs→Family resilience→Hope→Psychological resilience. The 95% CI of each path is (0.0157~0.335), (0.010~0.205), (0.015~0.146) and (0.019~0.125), respectively, all of which do not include 0, indicating that all the mediating effect paths exist. The detailed results are presented in **Table 5** and illustrated in **Figure 1**.

**Table 5:** Analysis of the mediation effects.

Model effect	Effect	Boot SE	BootLLCI	BootULCI	*Z*	*p*
Basic psychological satisfaction needs⇒Psychological resilience	0.098	0.046	0.157	0.335	2.141	0.032
Basic psychological satisfaction needs⇒Family resilience⇒Psychological resilience	0.042	0.049	0.010	0.205	0.857	0.391
Basic psychological satisfaction needs⇒Hope ⇒Psychological resilience	0.029	0.034	0.015	0.146	0.85	0.395
Basic psychological satisfaction needs⇒Family resilience⇒Hope⇒Psychological resilience	0.027	0.027	0.019	0.125	0.988	0.323

## 4 Discussion

This study reveals that the psychological resilience score of first-time AMI patients (62.95 ± 5.71) falls below normative thresholds. This result is significantly lower than that reported by N.L. et al. in their survey of general AMI patients (19). The discrepancy may stem from the exclusive inclusion of first-onset cases in our cohort. As an acute cardiovascular emergency, AMI often evokes a sense of impending doom, causing not only physical deterioration but also multifaceted stressors, such as decreased self-care ability, heavy economic burden and lack of various social roles (20). Confronted with these challenges, patients frequently demonstrate limited crisis management abilities, hindering effective coping strategies (21). Consequently, their psychological resilience levels remain suboptimal, exacerbating negative emotions and adversely affecting prognosis. It is suggested that clinicians prioritize psychological resilience assessments in first-time AMI patients to enhance mental health outcomes and facilitate better psychosocial adaptation.

Our correlation analysis shows that there is a positive correlation between CD-RIS and BPNS scores in patients with first-time AMI, and the total effect of basic psychological satisfaction needs on psychological resilience is significant (β=0.273, P < 0.001), demonstrating a positive predictive relationship. The degree of basic psychological need satisfaction is the performance of the individual's ability to participate in activities independently, show the ability to achieve goals and establish good relations with others (22). Previous studies confirm that patients with greater basic psychological need satisfaction exhibit enhanced subjective well-being, more positive emotional experiences, and improved social adaptation (23). Conversely, unmet basic psychological needs significantly increase risks for depression and other psychological/behavioral disorders (24, 25). Notably, robust psychological resilience buffers against adverse environmental influences, enabling patients to more readily achieve a high level of independent demand satisfaction, competent demand satisfaction and belonging demand satisfaction. Consequently, clinical nursing practice for first-time AMI patients should incorporate structured psychological support designed to improve disease-specific knowledge and rehabilitation awareness, which is essential for promoting basic psychological need satisfaction and facilitating positive adaptation to illness.

Mediating effect analysis of this study shows that basic psychological need satisfaction can positively predict family resilience and hope (β=0.489, 0.262, P < 0.001). At the same time, both family resilience and hope can positively predict patients' psychological resilience (β = 0.211, 0.273, P < 0.001). The results suggest that family resilience and hope constitute significant determinants of psychological resilience in this population. Critically, the effects of basic psychological need satisfaction, family resilience, and hope on psychological resilience function synergistically rather than independently (4). Hope level individuals tend to show stronger willpower, and such patients are more concerned about reality and willing to actively solve problems. Strong family resilience provides patients with stable emotional support, unconditional acceptance, and a collaborative problem-solving environment, which reduces their anxiety and fear and fosters a positive attitude toward the disease. Conversely, when family members experience poor psychological well-being, it can increase intra-family tension, thereby depleting the family's supportive resources. This shortage of resources makes it difficult for patients to receive adequate care and support, ultimately accelerating the decline in their hope levels (26–29). These results underscore the importance of integrating family-focused educational interventions into clinical nursing. Clinicians should encourage patients' families to provide robust emotional and practical support, as effective family assistance is crucial for promoting the patient's psychological well-being.

Further validation of mediation pathways confirms the significance of each path. Among them, the path of basic psychological satisfaction needs⇒family resilience⇒hope⇒psychological resilience has the highest clinical practicality. These findings indicate that basic psychological need satisfaction ultimately enhances psychological resilience through two synergistic mechanisms: strengthening family support systems and cultivating individual hope (30). Therefore, in clinical intervention, it is suggested to adopt various methods such as group psychological counseling, online and offline health education, and continuing nursing to meet patients' basic psychological needs as much as possible, and to improve family resilience and hope level by means of synchronous family intervention and peer education, so as to systematically enhance patients' psychological resilience in coping with first-time AMI.

However, this study is limited by its cross-sectional design, which only captures the participants' status at a single time point and cannot delineate their dynamic changes over time. Future research should employ longitudinal or intervention designs in first-time AMI patients to track the trajectory of psychological resilience and its influencing factors across different post-onset periods. Furthermore, the generalizability of the findings may be constrained by the relatively small sample size. Future studies with larger, more diverse samples and higher levels of evidence are warranted to further validate the effects of family resilience and hope on satisfying basic psychological needs and fostering psychological resilience.

## 5 Conclusion

This study analyzes the complex interaction among the basic psychological satisfaction needs, psychological resilience, family resilience and hope level of patients with first-time AMI. The results show that family resilience and hope serve as mediators between basic psychological need satisfaction and psychological resilience in patients with first-time AMI. Clinical care should coordinate community and family resources to enhance external social support and psychosocial companionship, so as to improve patients' psychological resilience and ultimately promote better disease prognosis.

## Data Availability

The original contributions presented in the study are included in the article/supplementary material. Further inquiries can be directed to the corresponding author.

## References

[1] Arabadjian M Duberstein ZT Sperber SH Kaur K Kalinowski J Xia Y. Role of Resilience in the Psychological Recovery of Women With Acute Myocardial Infarction. J Am Heart Assoc. (2023) 12:e027092. doi: 10.1161/JAHA.122.027092, PMID: 37026542 PMC10227277

[2] Wang J Wu Y Zhou J Li S She L. Resilience and its influencing factors after emergency percutaneous coronary intervention in young and middle-aged patients with first acute myocardial infarction. Sci Rep-UK. (2024) 14:9507. doi: 10.1038/s41598-024-59885-9, PMID: 38664486 PMC11045793

[3] Troy AS Willroth EC Shallcross AJ Giuliani NR Gross JJ Mauss IB. Psychological Resilience: An Affect-Regulation Framework. Annu Rev Psychol. (2023) 74:547–76. doi: 10.1146/annurev-psych-020122-041854, PMID: 36103999 PMC12009612

[4] Wang Y Qiu Y Ren L Jiang H Chen M Dong C. Social support, family resilience and psychological resilience among maintenance hemodialysis patients: a longitudinal study. BMC Psychiatry. (2024) 24:76. doi: 10.1186/s12888-024-05526-4, PMID: 38279114 PMC10811847

[5] Imran A Tariq S Kapczinski F de Azevedo CT. Psychological resilience and mood disorders: a systematic review and meta-analysis. Trends Psychiatr Psy. (2024) 46:e20220524. doi: 10.47626/2237-6089-2022-0524, PMID: 36215270 PMC11332678

[6] Zábó V Csiszar A Ungvari Z Purebl G. Psychological resilience and competence: key promoters of successful aging and flourishing in late life. Geroscience. (2023) 45:3045–58. doi: 10.1007/s11357-023-00856-9, PMID: 37418098 PMC10643728

[7] Jones JM. Surviving While Black: Systemic Racism and Psychological Resilience. Annu Rev Psychol. (2023) 74:1–25. doi: 10.1146/annurev-psych-020822-052232, PMID: 36652304

[8] Thabet A Ghandi S Barker EK Rutherford G Malekinejad M. Interventions to enhance psychological resilience in forcibly displaced children: a systematic review. BMJ Glob Health. (2023) 8:e007320. doi: 10.1136/bmjgh-2021-007320, PMID: 36731918 PMC9896216

[9] Pietrek A Kangas M Kliegl R Rapp MA Heinzel S van der Kaap-Deeder J. Basic psychological need satisfaction and frustration in major depressive disorder. Front Psychiatry. (2022) 13:962501. doi: 10.3389/fpsyt.2022.962501, PMID: 36203824 PMC9530199

[10] Li J. Longitudinal interplays between basic psychological need satisfaction and sleep among older adults in China. Soc Sci Med. (2023) 323:115862. doi: 10.1016/j.socscimed.2023.115862, PMID: 36965203

[11] Dou F Wang Q Wang M Zhang E Zhao G. Basic psychological need satisfaction and aggressive behavior: the role of negative affect and its gender difference. PEERJ. (2023) 11:e16372. doi: 10.7717/peerj.16372, PMID: 38025685 PMC10676081

[12] Kuang Y Wang M Yu NX Jia S Guan T Zhang X. Family resilience of patients requiring long-term care: A meta-synthesis of qualitative studies. J Clin Nurs. (2023) 32:4159–75. doi: 10.1111/jocn.16500, PMID: 36030397

[13] Richardson AL. Hope and anxiety. Curr Opin Psychol. (2023) 53:101664. doi: 10.1016/j.copsyc.2023.101664, PMID: 37572550

[14] Thygesen K Alpert JS Jaffe AS Chaitman BR Bax JJ Morrow DA. Fourth Universal Definition of Myocardial Infarction. Circulation. (2018) 138:e618–51. doi: 10.1161/CIR.0000000000000617, PMID: 30571511

[15] Sharif-Nia H Sánchez-Teruel D Sivarajan FE Hejazi S Hosseini L Khoshnavay FF. Connor-Davidson Resilience Scale: a systematic review psychometrics properties using the COSMIN. Ann Med Surg. (2024) 86:2976–91. doi: 10.1097/MS9.0000000000001968, PMID: 38694299 PMC11060289

[16] Kermavnar T Avsec A Huang S Desmet P. Assessing basic/fundamental psychological need fulfillment: systematic mapping and review of existing scales to foster cumulative science. Front Psychol. (2024) 15:1427478. doi: 10.3389/fpsyg.2024.1427478, PMID: 39403240 PMC11471629

[17] Nadrowska N Błażek M Lewandowska-Walter A. Polish adaptation of the Family Resilience Assessment Scale (FRAS). Community Ment Hlt J. (2021) 57:153–60. doi: 10.1007/s10597-020-00626-3, PMID: 32378127 PMC7813718

[18] Gao W Yuan C Wang J Du J Wu H Qian X. A Chinese version of the City of Hope Quality of Life-Ostomy Questionnaire: validity and reliability assessment. Cancer Nurs. (2013) 36:41–51. doi: 10.1097/NCC.0b013e3182479c59, PMID: 22495499

[19] Liu N, Liu S Yu N Peng Y Wen Y Tang J Kong L. Correlations among Psychological Resilience, Self-Efficacy, and Negative Emotion in Acute Myocardial Infarction Patients after Percutaneous Coronary Intervention. Front Psychiatry. (2018) 9:1. doi: 10.3389/fpsyt.2018.00001, PMID: 29410632 PMC5787139

[20] Swoboda C Gellert P Steinhagen-Thiessen E Landmesser U Rapp M Düzel S. Depression, anxiety, posttraumatic stress disorder and perceived psychosocial care during hospital stay after myocardial infarction: a cross-sectional study. BMC Cardiovasc Disord. (2025) 25:650. doi: 10.1186/s12872-025-05129-1, PMID: 40926210 PMC12418660

[21] Dai Q Kyuragi Y Zakia H Oishi N Yao L Zhang Z. Psychological resilience is positively correlated with Habenula volume. J Affect Disord. (2024) 365:178–84. doi: 10.1016/j.jad.2024.08.012, PMID: 39151760

[22] Wouters-Soomers L Van Ruysseveldt J Bos A Jacobs N. An individual perspective on psychological safety: The role of basic need satisfaction and self-compassion. Front Psychol. (2022) 13:920908. doi: 10.3389/fpsyg.2022.920908, PMID: 36059778 PMC9434267

[23] Shin H Park C. Social support and psychological well-being in younger and older adults: The mediating effects of basic psychological need satisfaction. Front Psychol. (2022) 13:1051968. doi: 10.3389/fpsyg.2022.1051968, PMID: 36507030 PMC9733472

[24] Lau S Connor LT Baum CM. Associations Between Basic Psychological Need Satisfaction and Motivation Underpinning Daily Activity Participation Among Community-Dwelling Survivors of Stroke: An Ecological Momentary Assessment Study. Arch Phys Med Rehab. (2023) 104:229–36. doi: 10.1016/j.apmr.2022.07.011, PMID: 35934048

[25] Cui P Shi J Li S Getu MA Wang R Chen C. Family resilience and its influencing factors among advanced cancer patients and their family caregivers: a multilevel modeling analysis. BMC Cancer. (2023) 23:623. doi: 10.1186/s12885-023-11101-z, PMID: 37403053 PMC10320962

[26] Ashley A Marsh K Ling J Lehto RH Wu HS Moser JS. Family Resilience in Adult Oncology: A Systematic Review and Meta-Analysis. Oncol Nurs Forum. (2025) 52:228–40. doi: 10.1188/25.ONF.228-240, PMID: 40293936 PMC12056869

[27] Bialek K Sadowski M Adamczyk-Gruszka O Mlodawski J Swiercz G. Basic hope, level of stress and strategies used to cope with stress after miscarriage during hospitalization and 3 months after its completion. Ginekol Pol. (2023) 95:22–31. doi: 10.5603/gpl.96215, PMID: 37994813

[28] Zhu Y Hua H Sheng L Zhou J Ye L Gu S. Relationship between disease perception and feelings of powerlessness in lymphoma patients: the mediating effect of social support and level of hope. Front Psychiatry. (2025) 16:1557867. doi: 10.3389/fpsyt.2025.1557867, PMID: 40084059 PMC11904247

[29] Deniz ME Satici SA Okur S Satici B. Relations among self-control, hope, and psychological adjustment: A two-wave longitudinal mediation study. Scand J Psychol. (2023) 64:728–33. doi: 10.1111/sjop.12927, PMID: 37243344

[30] Rochmawati E Berliana FR Wisdaningrum HO Paramastri A. Patient-reported hope and its predicting factors in malignant and non-malignant chronic illness: a cross-sectional study. Int J Palliat Nurs. (2024) 30:664–70. doi: 10.12968/ijpn.2024.30.12.664, PMID: 39688862

